# Remarks on a Case of an Acephalous Monster

**Published:** 1845-11

**Authors:** J. E. Manlove

**Affiliations:** Davidson county, Tennessee; Nashville


Art. IV.—*Remarks on a Case of an Acephalous Monster.* Read before the Medical Society of Tennessee at its Annual Meeting, on Thursday, 2d May, 1844. By J. E. Manlove, M.D., of Davidson county, Tenn.

On the 2d of January, 1842, I was requested to visit Mrs. L——e, a highly respectable and intelligent lady of my neighborhood, who supposed herself to be in labor with her sixth child. I found her laboring under a great deal of anxiety and solicitude, not so much on her own account, as from apprehensions that something was "wrong," as she said, about the child.

She stated that about the 4th month of pregnancy, one of her little sons, while playing about the yard, fell, and striking the hinder part of his head against a hard substance (perhaps an iron wedge) received a severe wound which bled very freely; that she was very much alarmed, and from the moment of the occurrence, up to that time, a period of nearly five months, she had occasionally had strange and unaccountable feelings—such as she had not experienced in any of her previous pregnancies.

I endeavored to compose and quiet her fears by giving her assurances that feelings such as she had described were not unusual; and that they had no connexion with the cause to which she ascribed them.

Finding that she was not in labor, I prescribed an anodyne for the night, and left her, with directions to be sent for again should circumstances require it.

On the following evening I was again sent for to see my patient, with whom I spent the night; her condition pretty much as at first visit. Great depression of spirits, with occasional slight pains complained of in the pelvic region, not such, however, as could be recognized as the pains of labor; at her suggestion, and more for her gratification, than from any belief on my part of its necessity, an examination *per vaginam* was made. The uterine tumor being very low down, was readily reached by the finger; cervix uteri obliterated; os tincæ tightly closed. From the peculiarly thin and attenuated feel of the walls of the uterus, and other symptoms, which usually precede and indicate labor, I had no hesitation in renewing my assurances of a speedy and favorable termination of her sufferings, both of body and mind, the latter of which had borne heavily upon her for the last five months.

Nothing in her condition forbidding it, I gave her half a grain sulph. morphia, which secured her a tolerable night's rest, and finding her free of pain in the morning, I again took my leave of her. But, as I expected, I was again summoned, in the course of twelve hours, to visit the patient—the messenger informing me that she was flooding, and that the family felt much alarm for her safety. As no time was to be lost, in three-quarters of an hour I was again at the dwelling of the patient. I was met at the door by an old mother, who occasionally officiates as midwife, who informed me that Mrs. L. was "very bad;" that the waters had suddenly broken off an hour and a half before my arrival; that shortly afterwards she had pains, with occasional slight discharges of blood, and sick fainty spells, and that she believed the after-birth was coming foremost.

I directed the old lady to prepare a little hot toddy while I proceeded to make an examination of the patient, who appeared then to be in a state of syncope. The finger was accordingly passed into the vagina, the upper two-thirds of which was partially occupied by a substance, which I entertained no doubt was the placenta, not only from the fact of hemorrhage, but also from its peculiar fibrous feel. Passing the finger still further within the os tincæ, which was well dilated and offered no resistance, it encountered a small, firm, globular body, apparently about the size of a large walnut; before I had time to satisfy myself of the character of the presentation, a contraction of the uterus coming on, expelled my hand, placenta, and child.

I found immediately upon its birth, that I had been mistaken in regard to the placenta, and continued my attention to the mother, until the after-birth was removed, and she secured from the danger of hemorrhage. Being apparently unconscious, she was not aware of the birth of the child, until the fact was communicated to her. Her first enquiry was, is not something the matter with the head? Being answered negatively, she asked to see it, which, it was thought, prudent to refuse, until she should more fully recover. She replied, despondingly, that she always knew something was the matter, and our refusal to let her see it confirmed her belief.

She did well, and is at this time in the enjoyment of good health. And now of the child, or rather monster, which is perhaps the more appropriate appellation. It did not breathe that I could discover, though there were visible movements of its limbs, such perhaps as are peculiar to intra-uterine life; it weighed two and a quarter pounds; measured eleven and a half inches in length, and four and a quarter across the shoulders. The inferior extremities, genital organs, trunk, and superior extremities, are proportioned, natural, and well developed. What gives interest to this singular specimen of monstrosity is the fact, that it is deficient in cervical vertebræ, as well as the bones at the sides and upper part of the cranium, and consequently in brain, and the membranes which ordinarily cover it. It has no forehead, but the skull runs backwards from the superciliary ridge, the eyes, remarkably large, when compared with the size of the face; the ears folding upon themselves, have the appearance of having been trimmed or foxed; and as it has no neck, they are almost in contact with the points of the shoulders.

That which I supposed during labor to be the placenta, proved to be a morbid growth, which leaving its attachment to the base of the skull, hung pendulous half way down the back of the child; it resembled very much a coagulum of blood or a piece of liver, with its thin edge down; it was covered with a thin membrane, which seemed to be a continuation of the common integuments; bled very freely upon moving or handling it; had a fibrous feel, and as it was the presenting part, was well calculated to induce an erroneous diagnosis.

Having finished my very imperfect description of this extraordinary formation, an enquiry very naturally suggests itself to the mind: Could the sight of the wound upon the occiput of the little boy, which, as we have already seen, gave the mother a great deal of uneasiness, have had any agency in this monstrous production?

It would be impossible to convince the mother, at least, to the contrary; and though we are not believers in ghosts and hobgobblins more than other people, there are circumstances about this case, which, viewing them as we do, have produced upon our mind impressions which cannot be forgotten.

That similar cases of monstrosity are recorded which could have been influenced by no such agency, I am fully aware. But does this fact prove that, because cases have occurred without any apparent cause, this particular case might not have been influenced by the circumstances which surrounded the mother, and which have already been detailed.

Dr. Ramsbotham in his valuable work on the Process of Parturition, says, in regard to this singular and interesting variety of deficient organization, that the acephalous monster is by no means of rare occurrence, that specimens may be seen in all the British museums—they prove that the brain is not essential to our being while in utero—that many of them have arrived at the full intra-uterine size, that some are actually larger than the ordinary fœtus, as if nature had intended to compensate for the brain by allowing an exuberant growth of the body. In these instances, continues our author, the nerves are well formed, and even those of the senses which ordinarily terminate in the cerebral mass itself—such as the optic—are not wanting, and many of them have been known to live for hours and even for days.

Nashville, May, 1845.

